# Generalist genes analysis of DNA markers associated with mathematical ability and disability reveals shared influence across ages and abilities

**DOI:** 10.1186/1471-2156-11-61

**Published:** 2010-07-05

**Authors:** Sophia J Docherty, Yulia Kovas, Stephen A Petrill, Robert Plomin

**Affiliations:** 1 King's College London, MRC Social, Genetic and Developmental Psychiatry Centre, Institute of Psychiatry, De Crespigny Park, London, SE5 8AF, UK; 2 Department of Psychology, Goldsmiths, University of London, New Cross, London, UK; 3 Center for Developmental and Health Genetics, The Pennsylvania State University, USA

## Abstract

**Background:**

The Generalist Genes Hypothesis is based upon quantitative genetic findings which indicate that many of the same genes influence diverse cognitive abilities and disabilities across age. In a recent genome-wide association study of mathematical ability in 10-year-old children, 43 SNP associations were nominated from scans of pooled DNA, 10 of which were validated in an individually genotyped sample. The 4927 children in this genotyped sample have also been studied at 7, 9 and 12 years of age on measures of mathematical ability, as well as on other cognitive and learning abilities.

**Results:**

Using these data we have explored the Generalist Genes Hypothesis by assessing the association of the available measures of ability at age 10 and other ages with two composite 'SNP-set' scores, formed from the full set of 43 nominated SNPs and the sub-set of 10 SNPs that were previously found to be associated with mathematical ability at age 10. Both SNP sets yielded significant associations with mathematical ability at ages 7, 9 and 12, as well as with reading and general cognitive ability at age 10.

**Conclusions:**

Although effect sizes are small, our results correspond with those of quantitative genetic research in supporting the Generalist Genes Hypothesis. SNP sets identified on the basis of their associations with mathematical ability at age 10 show associations with mathematical ability at earlier and later ages and show associations of similar magnitude with reading and general cognitive ability. With small effect sizes expected in such complex traits, future studies may be able to capitalise on power by searching for 'generalist genes' using longitudinal and multivariate approaches.

## Background

Quantitative genetic research indicates that individual differences in mathematical ability and disability are in part due to genetic factors, yielding heritability estimates of 0.2-0.9 [1-10]. In recent years quantitative genetic research has gone beyond the estimation of heritabilities to provide a deeper insight into the etiology of mathematics. The goal of the present paper is to address one of the most far-reaching recent theories emerging from such work; the Generalist Genes Hypothesis [11]. This hypothesis is based upon consistent multivariate genetic findings which suggest substantial genetic overlap between diverse learning abilities and disabilities. Multivariate genetic techniques analyze the genetic contribution to the phenotypic covariance among traits, and yield a statistic called the 'genetic correlation', which conveys the probability that a gene influencing one trait will also influence another. In the Twins Early Development Study (TEDS) sample, upon which the current report is based, an average genetic correlation of 0.84 was estimated for the performance of 10-year-olds on three diverse components of a web-administered mathematics test [12], suggesting that genetic effects are general across these components of mathematical ability. Furthermore, genetic effects on mathematics are also general across age. Despite cognitive and curriculum changes across middle childhood, longitudinal analysis has revealed genetic continuity across ages, with genetic correlations of 0.62-0.73 estimated between mathematical ability at ages 7, 9 and 10 [13].

Most surprisingly, genetic effects on mathematical ability appear even more general, extending to other learning and cognitive abilities [1,5,14-16]. For example, multivariate genetic analyses at 10 years of age in the TEDS sample yielded a genetic correlation of 0.79 between National Curriculum teacher ratings of Mathematics and English; 0.76 between online tests of mathematics and general cognitive ability (g); and 0.52 between online tests of mathematics and reading comprehension [9]. Such high genetic correlations between different abilities support the Generalist Genes Hypothesis. That is, the results suggest that although the influence of some genes will be ability specific and age specific (because genetic correlations are less than 1.0), the majority of the genetic influence over traits such as mathematics will be general across cognitive abilities and across ages [17]. If correct, the Generalist Genes Hypothesis could drastically change the way in which we view ostensibly distinct learning abilities and disabilities, and the way in which we search for the genes that influence them.

Although the molecular genetic basis of mathematical ability has received relatively little attention compared to other cognitive abilities, a recent TEDS study nominated 43 SNP associations from a two-stage genome-wide scan, comparing the pooled DNA of 10-year-olds of high vs. low mathematical ability [18]. When tested for association in an individually genotyped sample spanning the entire distribution of mathematical ability, 10 of these 43 SNP associations remained nominally significant. Though effects were of the small size now expected in complex traits [19,20], when combined into a SNP set these 10 SNPs accounted for 2.9% of the sample variance in mathematical ability. Furthermore, when all 43 SNPs were combined into a set, they accounted for 3.2% of the variance in mathematical ability [18]. Now that these first QTL associations have been identified, they can be used to empirically investigate the testable hypotheses arising from quantitative genetic work.

In the present study, we used the SNPs previously associated with mathematics at age 10 to empirically test the Generalist Genes Hypothesis. Specifically we tested three aspects of the Generalist Genes Hypothesis: SNPs associated with general mathematics ability at age 10 are 1) also associated with three different components of mathematics ability; 2) also associated with mathematics ability at earlier (7 and 9 years) and later (12 years) ages; and 3) also associated with reading and general cognitive ability at age 10. Although effect sizes are small, our results concur with those of quantitative genetic research in supporting the Generalist Genes Hypothesis.

## Results

### Analyses of components within the domain of mathematics at age 10

Our original genome-wide association study of mathematics at age 10 used a composite measure comprised of both teacher ratings and online-test results. Both the teacher and test measures were themselves composites of three different components of mathematics. Here we asked the extent to which the SNP sets associated with this general mathematics score at age 10 are also associated with each of these different components, as predicted by the multivariate quantitative genetic research mentioned above. Unlike our original study of mathematics at age 10, we have corrected the composite score for sex and age at time of testing, and we have used a missing data option to fill in missing genotypes for the 43-SNP-set score. Furthermore, 41 new individuals have been added to the original sample. Despite these differences, the first column of Table 1 shows SNP associations with the mathematics general composite at age 10 that are similar to those in our original report [18]. This similar pattern of results can be seen for the three 'best' SNPs (rs11225308, rs363449, rs17278234), the 10-SNP set and the 43-SNP set.

**Table 1:** SNP and SNP-set associations with measures of mathematics at age 10.

			Web Measures								Teacher-rated Measures							
			
	Mathematics composite at 10		Web total		Understanding number		Non-numerical processes		Computation and knowledge		Teacher total		Using and applying		Number and algebra		Shapes, space & measures	
	**Cor**	**N**	**Cor**	**N**	**Cor**	**N**	**Cor**	**N**	**Cor**	**N**	**Cor**	**N**	**Cor**	**N**	**Cor**	**N**	**Cor**	**N**

rs11225308	0.074**	2184	0.077**	1723	0.064**	1723	0.072**	1723	0.066**	1723	0.055**	1795	0.059**	1811	0.043*	1815	0.048*	1800

rs363449	0.069**	2182	0.070**	1703	0.057**	1703	0.041*	1703	0.075**	1703	0.067**	1802	0.070**	1820	0.064**	1823	0.063**	1807

rs17278234	0.067**	2188	0.050*	1708	0.059**	1708	0.038*	1708	0.033	1708	0.062**	1805	0.052*	1823	0.065**	1826	0.064**	1810

10-SNP set	0.167**	1888	0.145**	1486	0.151**	1486	0.107**	1486	0.119**	1486	0.145**	1551	0.132**	1567	0.146**	1571	0.141**	1556

43-SNP set	0.164**	1893	0.149**	1490	0.144**	1490	0.113**	1490	0.127**	1490	0.145**	1553	0.134**	1569	0.145**	1573	0.140**	1558

*r*_*p*_	NA	NA	0.911**	1854	0.822**	1845	0.714**	1854	0.822**	1854	0.915**	1933	0.872**	1967	0.880**	1970	0.876**	1957

Results of the tests of association of components of the mathematics composite at age 10 with rs11225308, rs363449, rs17278234, and the 10- and 43-SNP sets previously associated with the mathematics composite score. Associations are presented as correlations between the quantitative traits and additive genotypic values (as 0, 1, and 2 'increasing' alleles) for SNPs and additive SNP scores for SNP sets. Mathematics composite at 10 = the originally associated measure of mathematics at age 10; Web total = total web-test score at age 10; Understanding Number, Non-numerical Processes and Computation and Knowledge = three components of the web-test; Teacher total = teacher rating of mathematics at age 10; Using and Applying, Number and Algebra and Shapes, space and measures = three components of the teacher rating; Cor = Pearson product-moment correlations (p-values included in italics); N = size of sample analysed; *r*_*p *_= phenotypic correlation with mathematic composite score at age 10. One-tailed p-values were generated for all SNP-associations. * = nominally significant associations (p < 0.05). ** = associations withstanding Bonferroni testing for the five (three individual SNP and two SNP-set) association tests conducted simultaneously for each measure (p < 0.01).

Table 1 also displays the results of analyses involving the components of the mathematics composite score at age 10. As expected, high phenotypic correlations (> 0.7) are shown (see bottom row of results) between the composite score and each of its component test and teacher measures. It is not surprising then that the SNP-set scores previously associated with the mathematics composite are also highly significantly associated with all of these measures (all p-values < 1.8E-05). SNP associations are of a similar magnitude for online test scores and teacher ratings. Both SNP-set scores correlate significantly with the component measures of the online tests and teacher ratings but the magnitude of the correlations is more varied. For example, for the Non-Numerical Processes section of the web test, Table 1 shows correlations of 0.107 and 0.113 with the 10- and 43-SNP sets respectively, compared to correlations of 0.151 and 0.144 for the Understanding Number section. These small differences in correlations are not significant. Even greater variation is seen in the results of the single-SNP analyses of rs11225308, rs363449 and rs17278234, with the association of rs17278234 with the Non-Numerical Processes and the Computation and Knowledge sections of the web-test not reaching nominal significance (p < 0.05), and other single-SNP associations not standing up to Bonferroni correction for the five association analyses conducted on each measure (p < 0.01).

### Developmental analyses of mathematics at ages 7, 9 and 12

Mathematical ability was rated by teachers at ages 7, 9 and 12. At age 12, mathematical ability was also assessed through web-administered tests, testing the same three components assessed on the web at age 10. Table 2 displays the results of SNP-association analyses with these measures. 9-year mathematics scores yielded highly significant associations with both the 10- and the 43-SNP-sets, with correlations of 0.096 (p = 1.04E-04) and 0.090 (p = 2.84E-04) respectively, although these were not as strong as the correlations with the 10-year mathematics scores (Table 1). Individual SNP analyses of rs11225308, rs363449 and rs17278234 also yielded nominally significant associations, with those of rs11225308 and rs363449 remaining significant after correction for multiple testing. At 7 years, the association of the 10- and 43-SNP-sets with mathematics was significant but weaker still, both showing correlations of 0.046 (p = 0.004), and of the three individual SNPs tested, only rs17278234 was nominally associated.

**Table 2:** 10-year mathematics SNP and SNP-set associations with mathematical ability at ages 7, 9 and 12.

	Teacher mathematics at 7		Teacher mathematics at 9		Teacher mathematics at 12		Web mathematics at 12	
	Cor	N	Cor	N	Cor	N	Cor	N
rs11225308	0.019	3869	0.058**	1713	0.012	2213	0.025	3115
rs363449	0.016	3885	0.062**	1703	0.001	2223	0.018	3123
rs17278234	0.028*	3891	0.054*	1708	0.031	2227	-0.007	3132
10-SNP set	0.046**	3348	0.096**	1480	0.025	1922	0.036*	2691
43-SNP set	0.046**	3357	0.090**	1483	0.052*	1927	0.060**	2695
*r*_*p*_	0.594**	1979	0.633**	1632	0.566**	916	0.636**	1690

Association was assessed with rs11225308, rs363449, rs17278234, and the 10- and 43-SNP sets previously associated with mathematics at age 10. Associations are presented as correlations between the quantitative traits and additive genotypic values (as 0, 1, and 2 'increasing' alleles) for SNPs and additive SNP scores for SNP sets. Teacher mathematics at 7 = NC-based teacher-ratings of mathematical ability at age 7; Teacher mathematics at 9 = NC-based teacher-ratings of mathematical ability at age p; Web mathematics at 12 = performance on web-administered tests of mathematical ability at age 12; Teacher mathematics at 12 = NC-based teacher-ratings of mathematical ability at age 12; Cor = Pearson product moment correlations (p-values in italics); N = size of sample analysed; *r*_*p *_= phenotypic correlation with mathematic composite score at age 10. One-tailed p-values were generated for all SNP-associations. * = nominally significant associations (p < 0.05). ** = associations withstanding Bonferroni testing for the five (three individual SNP and two SNP-set) association tests conducted simultaneously for each measure (p < 0.01).

At 12 years, though none of the individual SNPs yielded significant associations, both SNP-sets were associated with online-test performance and the 43-SNP set was also associated with teacher ratings of mathematics - though only the association between the 43-SNP set and online-tests of mathematics at age 12 remained significant after Bonferroni correction. These results are in keeping with quantitative genetic findings demonstrating genetic continuity in the etiology of mathematical ability throughout the early school years. However, with comparable phenotypic correlations - ranging from 0.566 to 0.636 - between mathematical ability across these ages and the mathematics composite measure at age 10, the differences in association results shown in Table 2 also suggest some age specificity in the influence of these particular SNPs. This can be clearly seen in the individual-SNP analyses: Although sample sizes are large, there is very little association with mathematical ability at age 7 or 12.

### Analyses of associations with other learning and cognitive abilities

Reading and general cognitive ability were assessed at age 10 via online tests. Teacher ratings of reading ability at age 10 were also available. Table 3 displays the results from multivariate analyses of these measures. Significant associations were demonstrated with the 10- and 43-SNP sets, giving respective correlations of 0.121 (p = 2.2E-06) and 0.107 (p = 2.7E-05) for g, 0.118 (p = 1.3E-06) and 0.137 (p = 2.2E-08) for teacher-rated reading and 0.110 (p = 4.6E-06) and 0.101 (p = 2.6E-05) for the results of reading web-tests. Though single-SNP analyses of these 10-year measures revealed some nominal associations, after correction for multiple testing only those for web-test reading with rs17278234, and teacher-rated reading with rs11225308 and rs363449 remained significant.

**Table 3:** 10-year mathematics SNP and SNP-set associations with reading and general cognitive ability.

	g at 10		Teacher reading at 10		Web reading at 10	
	Cor	N	Cor	N	Cor	N
rs11225308	0.056*	1661	0.056**	1819	0.042*	1858
rs363449	0.032	1645	0.071**	1827	0.047*	1845
rs17278234	0.023	1650	0.033	1830	0.060**	1850
10-SNP set	0.121**	1431	0.118**	1576	0.110**	1609
43-SNP set	0.107**	1436	0.137**	1578	0.101**	1613
*r*_*p*_	0.596**	1762	0.673**	1971	0.517**	1953

Association was assessed with rs11225308, rs363449, rs17278234, and the 10- and 43-SNP sets previously associated with mathematics at age 10. Associations are presented as correlations between the quantitative traits and additive genotypic values (as 0, 1, and 2 'increasing' alleles) for SNPs and additive SNP scores for SNP sets. G at 10 = general cognitive ability at age 10; Teacher reading at 10 = NC-based teacher-ratings of reading ability at age 10; Web reading at 10 = performance on web-administered tests of reading at age 10; Cor = Pearson product moment correlations (p-values in italics); N = size of sample analysed; *r*_*p *_= phenotypic correlation with mathematic composite score at age 10. One-tailed p-values were generated for all SNP-associations. * = nominally significant associations (p < 0.05). ** = associations withstanding Bonferroni testing for the five (three individual SNP and two SNP-set) association tests conducted simultaneously for each measure (p < 0.01).

As one might expect from these multivariate results, and from those of previous multivariate quantitative genetic work, when we re-analysed the 10-year mathematics composite score with the 10-year measures of other cognitive abilities regressed out, the association was weaker. For example, respective correlations for the 10- and 43-SNP sets became 0.099 (p = 9.4E-05; N = 1431) and 0.118 (p = 3.9E-06; N = 1436) when 10-year g was controlled for, 0.117 (p = 1.8E-06; N = 1569) and 0.138 (p = 1.9E-08; N = 1573) when performance on 10-year reading web-tests was controlled for, and 0.075 (p = 0.002; N = 1431) and 0.096 (p = 1.4E-04; N = 1436) when both of these measures were controlled for. Though these correlations are indeed weaker, they remain significant, suggesting some specificity in the influence of these SNP sets to mathematics. In fact, even when g, web-test reading scores and teacher-rated reading scores were all controlled for - greatly restricting the size and power of the sample - the correlations with the 10- and 43-SNP sets were 0.046 (p = 0.060; N = 1139) and 0.072 (p = 0.007; N = 1141), respectively.

## Discussion

The results of our investigation into SNPs associated with mathematical ability are compatible with those of similar studies of SNPs associated with reading [21] and g [22], in providing strong molecular genetic support for the Generalist Genes Hypothesis which has emerged from quantitative genetic research. We have demonstrated that SNP sets identified on the basis of their association with a composite measure of mathematics at age 10 correlate significantly with each of the three diverse components of mathematics assessed both by web-based tests and teacher ratings. We have shown that the influence of these SNP sets on mathematics can be seen as early as age 7 and continues to age 12, and that these associations, which become weaker further away from the original target age, follow the simplex pattern expected from quantitative genetic longitudinal results [9,23]. The strongest support for the Generalist Genes Hypothesis comes from the mathematics SNP-set associations with reading and general cognitive ability at age 10.

Although we demonstrate overlap in the influence of the mathematics SNP sets with other cognitive abilities, our results also provide some evidence for genetic specificity. For example, when we regressed out variance due to both web-tested and teacher-rated reading scores at age 10 as well as variance due to age-10 general cognitive ability, the SNP sets still correlated with age-10 mathematical ability: 0.046 (p = 0.060; N = 1139) and 0.072 (p = 0.007; N = 1141) with the 10- and 43-SNP sets, respectively. Although the 10-SNP-set correlation is no longer significant, the 43-SNP set still accounts for 0.52% of the variation in mathematical ability at age 10 in our sample.

It would be interesting to know if this specificity was due to the specific action of particular SNPs. We individually analysed the three SNPs most strongly associated with mathematics at age 10 - rs11225308, rs363449 and rs17278234. Our analyses indicate that all three of these SNPs are associated with the other cognitive traits, though admittedly to varying degrees. Such a general influence may be expected, as the most proximal genes to these three SNPs - matrix metalloproteinase 7 (*MMP7*), glutamate receptor ionotropic kainate 1 (*GRIK1*) and dynein axonemal heavy chain 5 (*DNAH5*) - all play important yet quite general roles in development. Although *MMP7 *is involved in the breakdown of extracellular matrix during normal physiological processes such as embryonic development, growth and tissue repair [24], *GRIK1 *and *DNAH5 *have more direct links to the brain. *GRIK1 *encodes a glutamate receptor which mediates neurotransmission and synaptic plasticity [25,26], and dysfunction of this gene is implicated in a number of psychiatric phenotypes [27,28]. *DNAH5 *encodes the dynein axonemal heavy chain 5 protein, which is the force-generating component of cilia, the correct functioning of which is essential in all areas of embryonic growth [29]; *DNAH5 *in particular has been demonstrated as vital for normal brain development [30]. If *MMP7, GRIK1 *and *DNAH5 *underlie the SNP associations that we have reported, they may be indicative of the variety of 'generalist' genes involved in development - especially of the brain - which one might expect to exert small effects over a range of cognitive abilities.

In the present study, rs11225308, rs363449 and rs17278234 yielded correlations of 0.074, 0.069 and 0.067, respectively, with the 10-year mathematics composite (Table 1). With Ns of 2182 to 2188, power is marginal to detect such small correlations. However, the 10- and 43-SNP sets yielded correlations of 0.167 and 0.164 with the 10-year mathematics composite, and were significantly associated with many of the traits assessed here, even when the individual SNPs tested were not, which speaks to the value of using SNP sets. Moreover, despite containing 33 SNPs which failed to replicate their association with mathematics in the original study, the 43-SNP set performed just as well, and in some cases far better, than the 10-SNP set. Due to the nature of their selection - in a genome-wide association study bound by chance to generate some false positive results - it is unlikely that all of these 33 SNPs represent true associations. However, the superior performance of the 43-SNP set suggests that many of them may exhibit true effects on mathematics and other cognitive abilities that are too small to be detected when analysed alone. It also indicates that adding some false positive results into a composite SNP-set score containing a number of true positives is not detrimental to its performance in association analyses.

One of the main limitations of this study is its reliance on the same sample used to identify the 10-year mathematics SNPs. The TEDS sample, in which subjects have been assessed throughout development across a wide variety of traits, offers a prime opportunity for empirically testing the Generalist Genes Hypothesis at the molecular genetic level. However, if SNPs are associated with mathematics in this sample, it may not be surprising that they show associations with other traits with which mathematics is phenotypically correlated. Analyses conducted on two behavioural traits (data not shown) partially address this argument. Although measures of academic motivation and mathematics liking at age 12 correlate with mathematics at age 10 (0.356 and 0.448 respectively), they do not yield significant associations with the mathematics SNPs or SNP sets. Therefore, rather than simply reflecting phenotypic correlations between mathematics and other traits, our results seem to be more in line with the genetic correlations estimated in quantitative genetic research, supporting the Generalist Genes Hypothesis. Nevertheless, the fact that the cognitive measures assessed are correlated limits our ability to draw firm conclusions from this study. As all work on these SNPs has thus far been conducted within the TEDS sample, the replication of our findings in independent samples is essential before they can be generalised to the wider population.

A second limitation is the inclusion of individuals with missing genotype data in our 43-SNP-set analyses. As none of the 43 SNPs were in linkage disequilibrium, we were unable to impute the likely value of a missing genotype based upon available genotype information. Our substitution of missing genotypes with mean sample-scores did not affect our sample means, however it did artificially reduce variation. Though the use of a multiple imputation method [31] in forming a 43-SNP-set would have been complicated, the uncertainty associated with using missing values would have been more properly accounted for. Nonetheless, as the 43-SNP-set used here performed in much the same way as the 10-SNP-set, which did not include missing data, we would not expect multiple imputation to produce vastly different results. Our cross-sectional and univariate approach may also be seen as a limitation. As we assessed genetic associations in mathematical ability across several ages and abilities, it would have been possible to use longitudinal and multivariate models [32]. However, we suggest that the present focus on the Generalist Genes Hypothesis is better served by comparing associations across ages as well as across abilities, especially as the sample sizes often differ considerably.

Another possible limitation of this study lies with the measures used. Although quantitative genetic studies report genetic overlap between different cognitive abilities, and although we report an overlap in the association of specific genetic markers, this may be due to the complex nature of the tests employed. It is possible that the tests conventionally used to assess different learning abilities tap such a wide variety of cognitive processes that they will inevitably share many of the same genetic underpinnings. For this reason, it might be interesting to examine the association between the mathematics SNP sets and more specific cognitive processes. However, as there is evidence to suggest that even very specific tasks, such as different aspects of executive function [33] or information-processing measures [34], are highly genetically correlated, it is unlikely that we would see a significant deviation from the Generalist Genes perspective.

## Conclusions

In this report we have provided strong empirical support for the Generalist Genes Hypothesis, by demonstrating that SNPs previously associated with general mathematical ability at age 10 are also associated with different facets of mathematics, with mathematics at ages 7, 9 and 12, and with reading and general cognitive ability at age 10. Mathematical ability has only recently become the subject of molecular genetic investigation. As research continues and sample sizes grow, additional genetic markers will be uncovered, facilitating the formation of bigger and better SNP sets. To maximise power to detect small general effects, generalist gene approaches to the identification of such SNP sets could be used - allowing for the combination of samples assessed on different measures or aspects of mathematical ability, across different ages, or even of samples assessed on different learning abilities entirely. The identification of both general and trait- or age-specific genes associated with cognition, should lead to a greater understanding of the mechanisms involved in the development of diverse learning abilities and disabilities.

## Methods

### Participants

Participants were part of the Twins Early Development Study (TEDS), a longitudinal study involving a representative sample of over 11,000 sets of twins born in England and Wales between 1994 and 1996 [35,36]. The TEDS project has received ethical approval from The Joint South London and Maudsley and the Institute of PsychiatryResearch Ethics Committee (approval number: 05/Q0706/228), and the study of the genetics of mathematical cognition within the TEDS sample was approved by the King's College London Research Ethics Committee (PNM/07/08-47). Informed parental consent was obtained before all tests were conducted. Comparisons to UK census data show that the TEDS sample continues to be representative of the UK population in terms of demographic characteristics [37]. We excluded children with specific medical syndromes such as Down's syndrome and other chromosomal anomalies, cystic fibrosis, cerebral palsy, hearing loss, autism spectrum disorder, organic brain damage, extreme outliers for birth weight, gestational age, maternal alcohol consumption during pregnancy, special care after birth, non-white ethnic origin (to mitigate population stratification), English spoken as second language at home (to facilitate a fair comparison of test performance scores), and those without DNA samples available. Following this the sampling frame consisted of 4517, 4555 and 4562 children with genotypes available for rs11225308, rs363449 and rs17278234, respectively. The size of the sampling frames differ slightly here as the genotype data in our sample is incomplete, i.e. our sample contains missing genotypes. For the SNP-set analyses, the sampling frame consisted of 3919 children with complete genotype data on the 10 SNPs from the 10 SNP set, and 2895 children with complete genotype data on the 43 SNPs in the 43 SNP-set. However, as described below, for 1024 children with genotype data available on at least 40 of the 43 SNPs, we substituted the population mean for missing data, resulting in a total of 3929 children with 43-SNP-set data. From these sampling frames, all individuals possessing the relevant cognitive data were used in tests of association. As the amount of available data varies between the different cognitive measures assessed here - especially across ages - the N involved in each test of association also varies widely.

### SNP genotyping

As part of a recent genomewide association study of mathematical ability [18], the following 46 SNPs were selected from a high vs. low mathematical ability scan of pooled DNA in 10-year-old children, because they showed the most significant between-group differences: rs11225308, rs363449, rs17278234, rs11154532, rs12199332, rs12613365, rs6588923, rs2300052, rs6947045, rs1215603, rs40941, rs1881396, rs4649372, rs2593170, rs9300810, rs4314720, rs39118, rs694598, rs11778957, rs7085203, rs9670398, rs4956093, rs2278677, rs16964420, rs16907131, rs7932127, rs4236383, rs4144132, rs10098370, rs6502244, rs6701879, rs1502885, rs4771280, rs7791660, rs8043884, rs17085111, rs700965, rs7115849, rs1369458, rs7745469, rs952312, rs12962177, rs2059357, rs10501162, rs12601191 and rs696244. These SNPs progressed to the individual-genotyping stage of the study - where they were genotyped in a sample of 5000 TEDS subjects containing only one member of a twin-pair. At the time, only 2356 of these 5000 individuals possessed the relevant data for the original study of mathematics, however the current study of a number of traits is able to make use of the full sample. 41 SNPs were genotyped using the Sequenom MassARRAY iPlex Gold^® ^system (Sequenom, San Diego, USA), and 5 were genotyped using the Applied Biosystems' TaqMan^® ^assay (Applied Biosystems, California, USA). The medium-throughput Sequenom MassARRAY iPlex Gold^® ^system processes 'plexes' of up to 40 SNP-assays simultaneously. Only compatible assays may be combined into a single plex. Because of this, and to economise on cost and man-hours, the 41 SNPs investigated using the Sequenom iPlex Gold^® ^system were coupled with SNP-assays from other studies and spread across three plexes of 26, 33 and 36 SNPs. Individuals calling on fewer than 70% of the SNPs within each 'plex', and also within the TaqMan^®^-genotyped samples, were re-typed, as were SNPs with a call rate lower than 95%. 73 individuals with persistently low call-rates were removed entirely from the present study, leaving a sampling frame of 4927 individuals. However, on a 'within-plex' basis, 478, 434, 599 and 185 individuals were removed from the analysis of SNPs within the 26-plex, 33-plex, 36-plex and Taqman-genotyped SNPs, respectively. 3 SNPs with persistently low call rates were also removed: rs10501162, rs12601191 and rs696244. The 43 remaining SNPs were in Hardy-Weinberg equilibrium at the p > 0.01 level.

### SNP set

SNP-set scores have been used in a number of studies to aggregate the small effects of groups of SNPs [21,22,38,39]. This is especially useful in samples which may be underpowered to detect the effects of the SNPs when analysed separately. Two SNP-set scores were created for the current analysis. The first combines all 43 genotyped SNPs, and the second combines only the 10 SNP associations which replicated (p < 0.05) in the original study of mathematical ability (rs11225308, rs363449, rs17278234, rs11154532, rs12199332, rs12613365, rs6588923, rs2300052, rs6947045 and rs1215603). Using the direction of association observed in the original pooling stages, genotypes at each SNP were additively coded 0, 1 and 2 - with 0 representing the homozygote genotype associated with lower mathematical ability, 1 representing the heterozygote genotype, and 2 representing the genotype associated with higher mathematical ability. As none of the 43 SNPs are in linkage disequilibrium with one another, the additive genotypic scores should be independent. These genotypic scores were then summed to create a 10-SNP-set score of 0-20, and a 43-SNP-set score of 0-86. For the 10 SNP set, only the 3919 individuals with complete genotyping data were included in analyses. To increase the size and power of the sample to assess the influence of the 43-SNP set, 1024 individuals with at least 40 of the 43 genotypes available were included alongside the 2895 individuals with complete genotype data across all 43 SNPs, with the sample mean substituted for each missing genotype - giving a total of 3929 individuals with the 43-SNP-set score. As none of our SNPs were in linkage disequilibrium, and as estimates based upon mathematics scores or other genotypes may have biased our results, we could not impute missing data. By substituting missing genotypes with the mean genotypes for that SNP within our sample, we were able to analyse the available genotype data on at least 40 SNPs each for 1024 extra individuals, without affecting the mean genotype score of any of our SNPs.

### Measures

The validity and reliability of the composite measures of ability used in the present study, along with the testing procedures involved, have been described in detail previously [see [9,40]]. All web-based tests were undertaken in the participants' homes. Where internet access was unavailable in the home, participants were encouraged to use a school or library computer.

### Mathematics

#### National Curriculum teacher ratings of mathematics at ages 7, 9 and 10

Mathematical ability was measured by teachers' assessments on UK National Curriculum (NC) criteria for mathematical attainment at ages 7, 9 and 10 [41]. The National Curriculum is a framework used by all government-maintained schools across the UK to ensure that teaching and learning is balanced and consistent. NC-based ratings therefore provide a reliable and uniform measure of mathematical ability across our sample. Teacher assessments have been shown to be valid measures of academic achievement, particularly for mathematics, reading and language [42]. The teachers assessed three aspects of mathematical ability: Using and applying mathematics; Numbers and algebra; and Shapes, space and measures (see [9] for further details). In addition to using these components individually, we created a composite mean score by summing standardized scores for the three ratings.

### Web-based tests of mathematics at ages 10 and 12

The merits of web-based approaches have been well documented and findings appear consistent with traditional methods of data collection [43]. The battery used at ages 10 and 12 in this study included questions from three components of mathematics: 'Understanding Number', 'Computation and Knowledge' and 'Non-Numerical Processes' [12] (see Supplementary Materials for a more detailed description). These components correspond to the UK National Curriculum and thus increase the relevance of the study to education. Battery items were based on the National Foundation for Educational Research 5-14 Mathematics Series, which is linked closely to curriculum requirements in the UK and the English Numeracy Strategy [44]. In addition to analysis of each component, the results across the three components were standardised and then combined to generate a composite score.

### Composite score of mathematics at age 10

A composite mathematics score was generated from teacher-ratings and web-test results at 10 years of age following the same method used in the recent molecular genetic study of the trait [18]. Each measure was standardised to a mean of zero and standard deviation of one. For the 2976 TEDS children with data available the mean of the two measures was then standardised to form the composite score. For an additional 1106 children, only teacher ratings were available and for 942 children only web-based measures were available. To increase power, these children were also included in the original study, with their one available score standardised to a mean of zero and standard deviation of one.

### Reading at age 10

Reading performance was assessed at 10 years using both teacher assessments of achievement based on National Curriculum criteria, and the results of a web-based adaptation of the reading comprehension portion of the Peabody Individual Achievement Test (PIAT) [45] (See [9] for full details). The mean of the two standardised scores was also used as a composite reading measure.

### General cognitive ability at age 10

General cognitive ability was assessed at 10 years using web-based adaptations of two verbal tests - WISC-III Multiple (General Knowledge) choice Information and WISC-III Vocabulary Multiple Choice [46] - and two non-verbal reasoning tests - WISC-III-UK Picture Completion [46] and Raven's Standard Progressive Matrices [47]. Principal components analysis has revealed that the first principal component accounts for 55% of the variance in these measures at age 10 [9]. Thus, where results on all four tests were available, the four standardised scores were summed, and the mean standardised score was used as a composite measure of general cognitive ability.

### Statistical Analyses

All measures were standardised to a mean of 0 and a standard deviation of 1 and corrected for sex and age at time of testing. All analyses involved the estimation of Pearson product-moment correlations in R [48]. These were used to assess i) the phenotypic correlations of each of the cognitive measures with the composite measure of mathematical ability at age 10 ii) the association between each of the cognitive measures and the 10- and 43-SNP sets, and iii) the association between each of the cognitive measures and the three SNPs (included in both the 10- and 43-SNP sets) showing the strongest associations with the mathematics composite measure - rs11225308, rs363449 and rs17278234. In the original genome-wide association study of mathematical ability, the associations of these three SNPs remained after Bonferroni correction for multiple testing. Association was tested under the additive model by additively coding the genotypes of the individual SNPs and the SNP-set scores. One-tailed p-values are reported for all SNP analyses because we expect SNP associations in the same direction as previously observed with the composite measure of mathematics. P-values were also subject to Bonferroni correction for the five tests of association - with the two SNP sets and three individual SNPs - conducted simultaneously for each measure.

### Power

Power was estimated post-hoc using the Genetic Power Calculator [49]. The sample sizes involved in the present study varied greatly across measures. The mean N across all analyses was 2112. A sample of this size has 80% power to detect an association with an effect size of 0.45% (i.e a correlation of 0.067) for a causal QTL of 20% minor allele frequency. The smallest sample of 1431 individuals with data available on both the 10-SNP set and a measure of g at 10 has 80% power to detect an effect size of 0.7% (i.e. a correlation of 0.084). The largest sample of 3891 individuals with available data on both rs17278234 genotype and mathematics ratings at 7 years, had 80% power to detect an effect size of 0.25% (i.e. a correlation of 0.05).

## Authors' contributions

All authors were involved in the conception and design of the study. SJD conducted all analyses, interpreted results and drafted the manuscript. YK prepared the data, interpreted results and edited the manuscript for intellectual content. SAP and RP edited the manuscript for intellectual content.
